# Preceding Balance Disorders Affect Vestibular Function in Persistent Postural-Perceptual Dizziness

**DOI:** 10.3390/jcm12072589

**Published:** 2023-03-29

**Authors:** Mineko Oka, Kentaro Ichijo, Kento Koda, Teru Kamogashira, Makoto Kinoshita, Kazunori Igarashi, Takuya Kawahara, Ikumi Takashima, Tatsuya Yamasoba, Chisato Fujimoto

**Affiliations:** 1 Department of Otolaryngology and Head and Neck Surgery, Graduate School of Medicine, The University of Tokyo Hospital, Tokyo 113-8655, Japan; 2 Clinical Research Promotion Center, The University of Tokyo Hospital, Tokyo 113-8655, Japan

**Keywords:** dizziness, vestibular diseases, caloric tests, vestibular evoked myogenic potentials, persistent postural-perceptual dizziness, video head impulse test

## Abstract

Persistent postural-perceptual dizziness (PPPD) is induced by preceding conditions that cause balance disorders. To investigate the association between vestibular function and preceding balance disorders in PPPD patients, a retrospective chart review was performed. Vestibular function in 55 PPPD patients was measured using the caloric test, cervical vestibular evoked myogenic potential testing to air-conducted sound (ACS cVEMP), ocular vestibular evoked myogenic potential testing to bone-conducted vibration (BCV oVEMP), and video head impulse testing (vHIT). Patients were classified according to the type of preceding balance disorder. The age-stratified Cochran–Mantel–Haenszel (CMH) test and the exact test for the common odds ratio were conducted to evaluate the association between preceding n ≥ 4 balance disorders and present peripheral vestibular dysfunction. PPPD patients with preceding vestibular neuritis presented a significant positive association with abnormal caloric responses (*p* = 0.013), while those with preceding benign paroxysmal positional vertigo (BPPV) had significantly lower rates of abnormal BCV oVEMP (*p* = 0.003). Furthermore, patients with preceding vestibular neuritis showed lateral semicircular canal dysfunction, while those with preceding BPPV presented normal utricular functions. These results present the influence of preceding balance disorders on the vestibular function in PPPD.

## 1. Introduction

Persistent Postural-Perceptual Dizziness (PPPD) is a newly proposed clinical condition which presents a long-lasting sense of dizziness, defined by the Bárány Society in 2017 and included in the International Classification of Diseases by the World Health Organization in 2018 [1]. In PPPD, patients experience non-vertiginous dizziness for more than three months after a preceding balance disorder. The long-lasting sense of dizziness, which cannot be fully explained by preceding or comorbid balance disorders, is exacerbated by upright posture, active or passive movement, and exposure to moving or complex visual stimuli [1,2,3].

PPPD is the most common cause of chronic dizziness, which accounts for 12–19% of all dizziness patients [4,5]. In addition, PPPD-like chronic dizziness is reported to occur in approximately 25% of patients despite adequate compensation for or recovery from preceding acute vestibular disorders [6]. The most common balance disorders preceding PPPD are peripheral and central vestibular diseases such as BPPV or vestibular neuritis (25–30%), followed by attacks of vestibular migraine, psychological distress (panic attacks or anxiety), concussion or whiplash injuries, autonomic disorders, and a variety of other disorders [1,7,8].

Dizziness in PPPD persists even after the preceding balance disorder has resolved. Although there are no specific laboratory tests for PPPD, the balance function in PPPD patients may be influenced by a variety of different preceding disorders, leading to complex vestibular test results. Thus, in this study, we have investigated the effect of preceding balance disorders on vestibular function in PPPD patients to deepen the current knowledge of PPPD.

## 2. Materials and Methods

### 2.1. Ethical Statement

This study was approved by the research ethics committee of the Graduate School of Medicine and Faculty of Medicine, University of Tokyo (2487). The study was conducted according to the Declaration of Helsinki.

### 2.2. Subject

A retrospective chart review on patients who were diagnosed with PPPD at the Department of Otolaryngology and Head and Neck Surgery, the University of Tokyo Hospital from 2017 to 2021 was conducted. PPPD was diagnosed using the criteria from the Committee for Classification of Vestibular Disorders of the Bárány Society [1]. Patient age, sex, and any preceding or comorbid balance disorders were recorded. Vestibular function tests included caloric testing, cervical vestibular evoked myogenic potential testing to air-conducted sound (ACS cVEMP), ocular vestibular evoked myogenic potential testing to bone-conducted vibration (BCV oVEMP), and video head impulse testing (vHIT). All tests were performed on the same day for each patient.

### 2.3. Caloric Test

Caloric testing was performed to clinically assess the function of the lateral semicircular canal (LSCC) and superior vestibular nerve [9]. Patients were asked to take a supine position in a dark room while closing their eyes. All eye movements were recorded using electronystagmography. A 20 s injection of 2 mL ice water (4 °C) into the external auditory canal was performed to induce nystagmus. “Canal paresis (CP) percentage >20%” or “maximum slow phase eye velocity <10°/s for both ears” was considered abnormal.

### 2.4. ACS cVEMP Testing

ACS cVEMP testing was performed to clinically assess the function of the saccule and inferior vestibular nerve [10,11]. The recording method of ACS cVEMP has been previously described [12]. In brief, surface electromyographic (EMG) electrodes were placed at the center of each sternocleidomastoid muscle (SCM) and the reference electrode was placed on the lateral end of the upper sternum in a symmetric position. During the recording, patients were asked to sit with good posture and rotate the neck to stretch the SCM. A Neuropack MEB-2306 (Nihon Kohden Co. Ltd., Tokyo, Japan) was used to record cVEMP. Acoustic stimuli consisted of air-conducted 500 Hz short tone-bursts (135 dB SPL, rise/ fall time 1 ms, plateau time 2 ms) and were presented through headphones at a stimulation rate of 5 Hz. The signals were amplified and bandpass filtered (20–2000 Hz). The analysis time was 100 ms (20 ms before and 80 ms after the stimulus). Recordings were performed twice for each ear to confirm reproducibility. The amplitude and latency of the first positive–negative peak (p13–n23) was calculated as the average of the two responses. The p13–n23 amplitude recorded by the ipsilateral side stimulation was used for analysis. Corrected amplitude (CA) was calculated as the p13–n23 amplitude divided by the measure of SCM contraction. The asymmetry ratio (AR) for cVEMPs (cVEMP AR) was calculated as AR = 100 × (CAu − CAa)/(CAu + CAa), where CAu represents the CA on the unaffected side and CAa represents the CA on the affected side. An irreproducible p13–n23 was considered to be an absent response. A value for cVEMP AR greater than the normal upper limit, which was set at 41.6, was considered to be a decreased response [13]. Absent cVEMP responses on both sides measured by SCM contraction were considered to be bilateral abnormal cVEMPs.

### 2.5. BCV oVEMP Testing

BCV oVEMP testing was performed to evaluate the function of the utricle and the superior vestibular nerve [14,15]. Recording methods for BCV oVEMPs have been previously described [12]. In brief, surface electrodes were placed on the skin 1 cm below (active) and 3 cm below (reference) the center of each lower eyelid. During the recording, patients were asked to sit and look upward about 30°. A Neuropack MEB-2306 was used to record oVEMPs. Bone-conducted stimuli consisted of 500 Hz tone-bursts (rise/fall time 1 ms, plateau time = 2 ms) and were delivered by a Type 4810 Mini-Shaker (Bruel and Kjaer, Naerum, Denmark) placed on the forehead on the midline (Fz), at a stimulation rate of 3 Hz. The driving voltage was adjusted to 8.0 V (peak to peak) to produce a peak force level of 128 dB re 1 mN. The signal was amplified and bandpass-filtered (0.5–500 Hz). The analysis time was 50 ms. Recordings were performed twice to confirm reproducibility. The amplitude and latency of the first negative–positive peak (n1–p1) was calculated as the average of the two responses. The n1–p1 amplitude recorded by the ipsilateral side stimulation was used for analysis. The asymmetry ratio for oVEMP (oVEMP AR) was used to evaluate the abnormality of the n1–p1 amplitude [16]. An irreproducible n1–p1 was considered to be an absent response. A value for oVEMP AR greater than the normal upper limit, which was set at 27.3, was considered to be a decreased response [17]. Absent oVEMP responses on both sides were considered to be bilateral abnormal oVEMPs.

### 2.6. vHIT

The vHIT was performed to assess the vestibulo-ocular reflex (VOR) in the three semicircular canal planes using an ICS Impulse (Otometrics, Taastrup, Denmark). Subjects were seated 1 m from a black fixation dot on a wall that served as the visual target. While the subject gazed at the fixation dot, the examiner briefly and unpredictably rotated the subject’s head. The head rotations were made in the lateral, the left anterior-right posterior, and the right anterior-left posterior planes. VOR gains were analyzed based on the manufacturer’s algorithms, using 175 samples out of a total of 250 samples obtained on each trial. Data from the onset of head motion and subsequent zero crossing of head velocity were used to measure the area under the curve (AUC) of head velocity. The value of VOR gain was calculated as (AUC of eye velocity)/(AUC of head velocity). A mean VOR gain of <0.7 for the vertical semicircular canal (VSCC) or <0.8 for the LSCC was considered to be abnormal function with vHIT [18].

### 2.7. Statistical Analysis

Statistical analyses were conducted using SAS software, version 9.4 (SAS Institute). Patient background factors were summarized by mean ± SD for continuous variables and by number and percentage constituent of patients for categorical variables. PPPD patients were classified according to the type of preceding balance disorder. A contingency table relating vestibular function tests and preceding balance disorder was created. Cochran–Mantel–Haenszel (CMH) tests stratified by age (≤40, 41–50, 51–60, 61–70, >70years) were performed to evaluate the association between preceding balance disorder with n ≥4 and the existence of peripheral vestibular dysfunction detected by caloric, ACS cVEMP, BCV oVEMP tests, and vHIT (bilaterally abnormal or unilaterally abnormal/normal) [19]. To complement the CMH test, which might possibly underestimate *p*-values due to the small number of patients, Zelen’s common odds ratio test, which provides *p*-values from exact probability calculations while stratifying patients by age [20], was performed in addition. A *p*-value < 0.05 was considered as statistically significant. The presence of an association between the preceding balance disorder and peripheral vestibular dysfunction was defined as statistically significant results with both the CMH and Zelen’s test.

## 3. Results

A total of 55 patients were diagnosed with PPPD. Of the 55 patients, 38 underwent all four vestibular function tests. Nine refused to take vHIT testing due to worsening of dizziness with head motion. Three did not undergo vHIT because of neck pain after head injury or whiplash based on concerns that the test may worsen their symptoms. Five did not undergo vHIT for VSCCs, because of the exacerbation of dizziness by passive head rotation during vHIT for LSCCs. One patient received only the caloric and BCV oVEMP test because of a prior cervical spine surgery for congenital scoliosis.

Characteristics of the 55 PPPD patients are shown in Table 1. Average patient age was 51.5 ± 14.8, ranging from 18 to 84 years old. Eleven were males and forty-four were females. Benign paroxysmal positional vertigo (BPPV), Ménière’s disease, head injury or whiplash, and vestibular neuritis was observed in eight, seven, six and four patients, respectively. In half of the cases with BPPV (four cases), the responsible semicircular canal could not be identified because the diagnosis was made at another hospital and the specific nystagmus had disappeared by the time the patient came to our institution. The other four cases consisted of two lateral and two posterior semicircular canal BPPV patients. Psychological stress, Ramsay Hunt syndrome, sudden sensorineural hearing loss, vertebrobasilar insufficiency, and delayed endolymphatic hydrops were present in two patient each. Cerebral hemorrhage, migraine, fever due to pyelonephritis and mal de debarquement were reported in one patient each. These preceding diseases were diagnosed at the clinic or hospital of origin. The remaining 16 patients developed PPPD after vertigo of unknown cause, among whom 11 had spontaneous and 5 had positional vertigo, respectively. These cases were not examined by an otolaryngologist when the preceding balance disorder had occurred before PPPD, or otherwise a detailed examination did not lead to a final diagnosis. The average duration of disease was 22.4 ± 36.2 months. Five patients had psychiatric disorders, all with depression, and an additional panic disorder and delusional disorder in one patient each. Five patients were taking sedatives or antidepressants, four of which contained benzodiazepines.

Abnormal caloric responses were observed in 20 patients (36%), with 19 patients presenting unilateral canal paralysis. Abnormal ACS cVEMPs were observed in 34 patients (62%). Among the 34 patients with abnormal ACS cVEMPs, 14, 14, and 6 patients presented bilateral absent responses, unilateral absent responses, and unilateral decreased responses, respectively. Abnormal BCV oVEMPs were observed in 22 patients (40%). Among the 22 patients with abnormal BCV oVEMPs, 3, 10, and 9 patients presented bilateral absent responses, unilateral absent responses, and unilateral decreased responses, respectively. Abnormal vHITs for the LSCC were observed in 8 of 43 patients tested (19%). Among the 8 patients, 2 and 6 patients presented bilateral and unilateral abnormal functions, respectively. Abnormal vHITs for the anterior semicircular canal (ASCC) were observed in 14 of 38 patients tested (37%). Among the 14 patients, 6 and 8 patients presented bilateral and unilateral abnormal results, respectively. Abnormal vHITs for the posterior semicircular canal (PSCC) were observed in 16 of 38 patients tested (42%). Among the 16 patients, 6 and 10 patients presented bilateral and unilateral abnormal outcomes, respectively (Table 2).

First, we investigated whether each of the preceding diseases with *n* ≥ 4 (BPPV [*n* = 8], Ménière’s disease [*n* = 7], head injury/whiplash [*n* = 6], vestibular neuritis [*n* = 4], spontaneous vertigo [*n* = 11], positional vertigo [*n* = 5]) had an association with abnormalities in caloric testing (Table 3). PPPD patients with preceding vestibular neuritis presented a significantly positive association with abnormal caloric responses in both the CMH test (*p* = 0.013, CMH risk ratio 3.12, 95% confidence interval [CI] 1.49–6.55) and the exact test for the common odds ratio (*p* = 0.048, 95% CI, 1.47–∞), while other preceding diseases failed to show a significant result (Table 3).

Next, we investigated whether each of the preceding diseases with *n* ≥4 had an association with abnormalities in ACS cVEMPs. No association was found between preceding diseases and abnormalities in ACS cVEMPs (Table 3).

Then, we investigated whether each of the preceding diseases with *n* ≥4 had an association with abnormalities in BCV oVEMPs (Table 3). PPPD patients with preceding BPPV had significantly lower rates of abnormal BCV oVEMPs in both the CMH test (*p* = 0.003, CMH risk ratio 0) and the exact test for the common odds ratio (*p* = 0.004, 95% CI 0–0.34). However, those with preceding spontaneous vertigo of unknown cause had significantly lower rates of abnormal BCV oVEMPs with the CMH test (*p* = 0.037, CMH risk ratio 0.17, 95% CI 0.02–1.48), but not with the exact test for the common odds ratio. Other preceding diseases did not present any significant correlation with abnormalities in BCV oVEMPs.

Finally, we investigated whether each of the preceding diseases with *n* ≥4 had an association with abnormalities in vHITs (Table 3). Head injury or whiplash was excluded from the analysis because only three cases received vHITs. PPPD patients with preceding BPPV and spontaneous vertigo of unknown cause had significantly higher rates of abnormal vHIT results with the CMH test (*p* = 0.041, CMH risk ratio 3.31, 95% CI 1.12–9.78/*p* = 0.047, CMH risk ratio 0.22, 95% CI 0.03–1.46, respectively), but not with exact test for the common odds ratio. Other preceding diseases did not present any significant correlation with abnormal vHIT results (Table 3).

## 4. Discussion

In this study, we have revealed the association of vestibular function in PPPD with preceding balance disorders. Some previous articles have focused on the vestibular function in PPPD [5,21], including a few which have suggested specific laboratory findings for this disease [22,23]. For example, the functional head impulse test with optokinetic stimulation provoked more reading errors in patients with PPPD than in controls [22]. In addition, a significantly greater head-tilt perception gain in the head roll-tilt subjective visual vertical test was reported with PPPD compared to unilateral vestibular hypofunction and psychogenic dizziness [23]. Furthermore, phobic postural vertigo, a classical disease concept with similar clinical features to PPPD, has been reported to present worsening of staggering especially when viewing moving visual scenes, and increased sway in the 3.53–8 Hz frequency band [24,25,26]. However, to our knowledge, there have been no reports on the effect of prior balance disorders on the vestibular function of PPPD.

Here, we have found that the presence of preceding vestibular neuritis had a significantly positive association with abnormalities in caloric tests in PPPD patients, suggesting that vestibular dysfunction, especially dysfunction of the LSCC, is affected by this preceding disease. Vestibular neuritis is known to present hypofunction of the semicircular canal, which can be detected by the caloric test [27]. In accordance with this finding, all patients diagnosed with vestibular neuritis showed unilateral abnormal caloric response in our previous report [28]. Again, in this study, all PPPD patients with preceding vestibular neuritis showed unilateral abnormal caloric responses. It has been reported that patients who develop severe acute unilateral peripheral vestibular disorders suffer from postural instability even in the chronic phase and did not improve as much as normal subjects [29]. Even though some clinical symptoms in PPPD cannot be explained solely by preceding or other complicating diseases, persistent imbalance during upright posture is a symptom that is also observed in the chronic phase of peripheral vestibular disorders. Thus, our study suggests a residual effect of vestibular dysfunction caused by preceding peripheral vestibular diseases on persistent imbalance in PPPD. On the other hand, there was no significant association between the presence of vestibular neuritis and abnormalities in the LSCC vHIT. In vestibular neuritis, the abnormality rate of the caloric test was 100%, but that of the LSCC vHIT was only 50%. However, vHIT is reported to be less sensitive than caloric testing when assessing moderate vestibular dysfunction in vestibular neuritis patients [30]. Therefore, the difference in the abnormality rate between the two tests could be explained by partial recovery of vestibular function during a 3-month or longer duration which had elapsed from the onset of vestibular neuritis.

In addition, PPPD patients with preceding BPPV presented no oVEMP abnormalities, which was significantly lower than those with other preceding diseases. This reflects the stability of the utricle system in PPPD patients with preceding BPPV, suggesting a limited effect of BPPV on otolith function. In other words, compared to diseases such as vestibular neuritis, sudden sensorineural hearing loss, and Ramsay Hunt syndrome that strongly impair vestibular function, the damage caused by BPPV is relatively small. We have previously reported that 30% of BPPV patients presented abnormal oVEMPs, which increased with age [31]. Although the prevalence of abnormal oVEMPs is much lower in this study, this may be explained by the considerably younger age of PPPD patients with preceding BPPV (55.6 ± 18.4 years old) than those of BPPV patients in our previous literature described above (63.0 ± 14.2 years old).

Meanwhile, patients with preceding unexplained vertigo did not present a significant association with vestibular dysfunction even though more than half of the cases presented abnormal cVEMPs. These patients were not examined by an otolaryngologist during the primary balance disorder before PPPD and were unable to reach a final diagnosis. Their preceding balance disorders were left undiagnosed, or otherwise may include clinically unestablished diseases. Among the 16 patients, 5 patients presented positional vertigo, a brief vertigo during head rotation, similar to those of BPPV. As mentioned above, these patients are less likely to present vestibular dysfunction due to the limited effect of BPPV on otolith function. On the other hand, the other 11 patients showed spontaneous vertigo, including 3 who experienced recurrent vertigo attacks lasting as long as several hours, but without any cochlear symptoms. The three patients may have been diagnosed with vestibular Ménière’s disease (American Academy of Ophthalmology and Otolaryngology, 1972) or possible Ménière’s disease (American Academy of Otolaryngology Head and Neck Surgery, 1995) [32] if they had been referred to an otolaryngologist at that time. The other eight patients with spontaneous vertigo presented single or brief vertigo, which is unlikely to cause vestibular dysfunction. Taken together, although preceding unexplained vertigo in PPPD patients failed to show a significant correlation with residual vestibular dysfunction, most patients presented cVEMP abnormalities, which is in correspondence with previous literature reporting frequent vestibular dysfunction in patients without a specific vestibular disease diagnosis prior to PPPD [5].

This study had several limitations. First, information and selection bias cannot be excluded because this was a retrospective study. The latter may also arise since this study was conducted at a tertiary research institution specialized in vertigo and dizziness. Second, although assumptions could be made, we cannot draw a clear conclusion as to why preceding vestibular neuritis was associated with abnormalities in caloric tests but not with VEMP testing and vHITs. Third, preceding balance disorders prior to PPPD may include undiagnosed or clinically unestablished diseases. Fourth, the onset of detected vestibular dysfunction in this study remains unknown, because most preceding balance disorders were diagnosed elsewhere, lacking an assessment on vestibular function at that time. Fifth, preceding diseases in some patients may be persistent, acting as a comorbid vestibular disease with PPPD. Thus, we cannot fully differentiate the sequela of the preceding vestibular disease with disequilibrium caused by an active balance disorder. Last, there was a lack of data in vHIT analysis because of the exacerbation of dizziness caused by head motion, a common characteristic of PPPD.

Nonetheless, our study indicates an influence of preceding balance disorders on vestibular function in PPPD. This highlights the importance of closely monitoring vestibular function with caloric tests, VEMP testing, and vHITs, based on the type of preceding balance disorders.

## 5. Conclusions

PPPD with preceding vestibular neuritis showed dysfunction of the lateral semicircular canal, suggesting the influence of residual vestibular damage on PPPD. Meanwhile, PPPD with preceding BPPV did not present any dysfunction of the utricular system, suggesting a limited effect of BPPV on otolith function. These results present the influence of preceding balance disorders on vestibular function in PPPD. This highlights the importance of closely monitoring vestibular function with caloric tests, VEMP testing, and vHIT, based on the type of preceding balance disorders.

## Figures and Tables

**Table 1:** Characteristics of 55 persistent postural-perceptual dizziness patients.

Preceding Diseases	n (%)	Age (Years), Mean ± SD	Sex, Female n (%)	Disease Duration (Months), mean ± SD	Psychiatric Comorbidities, n (%)	Use of Sedative Drugs, n (%)
All patients	55	51.5 ± 14.8	44 (80%)	22.4 ± 36.2	5 (10%)	5 (10%)
BPPV	8 (15%)	56.3 ± 18.4	6 (75%)	13 ± 19.2	1 (13%)	1 (13%)
Ménière’s disease	7 (13%)	48.1 ± 11.3	5 (71%)	5.4 ± 3.6	1 (14%)	1 (14%)
Head injury/whiplash	6 (11%)	47.7 ± 21.6	6 (100%)	74.2 ± 83.6	1 (17%)	1 (17%)
Vestibular neuritis	4 (7%)	63.0 ± 10.4	4 (100%)	27.8 ± 31.9	1 (25%)	1 (25%)
Psychological stress	2 (4%)	25.0 ± 9.9	2 (100%)	8.0 ± 2.8		1 (50%)
Ramsay Hunt syndrome	2 (4%)	56.5 ± 16.3	2 (100%)	31.5 ± 40.3		
Sudden sensorineural hearing loss	2 (4%)	60.5 ± 6.4	1 (50%)	39.0 ± 12.7	1 (50%)	
Vertebrobasilar insufficiency	2 (4%)	68.0 ± 12.7	1 (50%)	5.5 ± 0.7		
Delayed endolymphatic hydrops	2 (4%)	53.0 ± 9.9	2 (100%)	3.0		
Cerebral hemorrhage	1 (2%)	60.0	0	48.0		
Migraine	1 (2%)	54.0	1 (100%)	15.0		
Fever due to pyelonephritis	1 (2%)	61.0	0	9.0		
Mal de debarquement	1 (2%)	72.0	1 (100%)	72.0		
Vertigo of unknown causes	16 (29%)	46.1 ± 9.2	13 (81%)	13.4 ± 12.4		
Spontaneous vertigo	11 (20%)	44.8 ± 9.3	9 (82%)	16.5 ± 14.0		
Positional vertigo	5 (9%)	48.8 ± 9.6	4 (80%)	6.8 ± 2.6		

PPPD = persistent postural-perceptual dizziness, BPPV = benign paroxysmal positional vertigo, SD = standard deviation. Disease duration indicates the duration of PPPD.

**Table 2:** Results of vestibular function tests in persistent postural-perceptual dizziness patients.

	Total	BPPV	Ménière’s Disease	Head Injury /Whiplash	Vestibular Neuritis	Vertigo of Unknown Causes	
						Spontaneous Vertigo	Positional Vertigo
	n = 55	n = 8	n = 7	n = 6	n = 4	n = 11	n = 5
**Caloric test**							
Bilaterally abnormal	1 (2%)	0 (0%)	1 (14%)	0 (0%)	0 (0%)	0 (0%)	0 (0%)
Unilaterally abnormal	19 (35%)	4 (50%)	2 (29%)	1 (17%)	4 (100%)	1 (9%)	0 (0%)
Normal	35 (64%)	4 (50%)	4 (57%)	5 (83%)	0 (0%)	10 (91%)	5 (100%)
**ACS cVEMPs**							
Bilaterally abnormal	14 (26%)	1 (14%)	1 (14%)	0 (0%)	1 (25%)	5 (45%)	1 (20%)
Unilaterally abnormal	20 (37%)	2 (29%)	2 (29%)	3 (50%)	1 (25%)	3 (27%)	1 (20%)
Normal	20 (37%)	4 (57%)	4 (57%)	3 (50%)	2 (50%)	3 (27%)	3 (60%)
Unexamined	1	1					
**BCV oVEMPs**							
Bilaterally abnormal	3 (5%)	0 (0%)	1 (14%)	1 (17%)	0 (0%)	0 (0%)	1 (20%)
Unilaterally abnormal	19 (35%)	0 (0%)	2 (29%)	2 (33%)	3 (75%)	1 (9%)	2 (40%)
Normal	33 (60%)	8 (100%)	4 (57%)	3 (50%)	1 (25%)	10 (91%)	2 (40%)
**vHIT (LSCC)**							
Bilaterally abnormal	2 (5%)	0 (0%)	0 (0%)	0 (0%)	0 (0%)	1 (13%)	0 (0%)
Unilaterally abnormal	6 (14%)	0 (0%)	1 (20%)	0 (0%)	2 (50%)	1 (13%)	0 (0%)
Normal	35 (81%)	7 (100%)	4 (80%)	4 (100%)	2 (50%)	6 (75%)	5 (100%)
Unexamined	12	1	2	2		3	
**vHIT (ASCC)**							
Bilaterally abnormal	6 (16%)	1 (17%)	1 (20%)	0 (0%)	1 (25%)	1 (14%)	1 (25%)
Unilaterally abnormal	8 (21%)	3 (50%)	1 (20%)	1 (33%)	1 (25%)	0 (0%)	1 (25%)
Normal	24 (63%)	2 (33%)	3 (60%)	2 (67%)	2 (50%)	6 (86%)	2 (50%)
Unexamined	17	2	2	3		4	1
**vHIT (PSCC)**							
Bilaterally abnormal	6 (16%)	1 (17%)	1 (20%)	0 (0%)	1 (25%)	1 (14%)	1 (25%)
Unilaterally abnormal	10 (26%)	3 (50%)	1 (20%)	1 (33%)	1 (25%)	0 (0%)	1 (25%)
Normal	22 (58%)	2 (33%)	3 (60%)	2 (67%)	2 (50%)	6 (86%)	2 (50%)
Unexamined	17	2	2	3		4	1
Variable distributions are reported as n (%).							

Abbreviations: PPPD = persistent postural-perceptual dizziness, BPPV = benign paroxysmal positional vertigo, ACS cVEMP = cervical vestibular evoked myogenic potential testing to air-conducted sound, BCV oVEMP = ocular vestibular evoked myogenic potential testing to bone-conducted vibration, vHIT = video head impulse test, LSCC = lateral semicircular canal, ASCC = anterior semicircular canal, PSCC = posterior semicircular canal.

**Table 3:** Cochran–Mantel–Haenszel test and exact test for the common odds ratio (Zelen) to examine the association between abnormalities in vestibular function tests and preceding disease in persistent postural-perceptual dizziness patients.

Variable	Risk Ratio (CMH)	95% CI (CMH)	*p*-Value (CMH)	95% CI (Zelen)	*p*-Value (Zelen)
**Caloric test**					
BPPV	1.14	0.46–2.84	0.778	0.20–7.73	1.000
Ménière’s disease	1.55	0.57–4.26	0.400	0.24–18.46	0.679
Head injury/whiplash	0.31	0.05–2.04	0.144	0.00–2.39	0.313
Vestibular neuritis	3.12	1.49–6.55	0.013 *	1.47–∞	0.048 *
Spontaneous vertigo	0.28	0.04–1.85	0.113	0.00–1.84	0.232
Positional vertigo	0	-	0.151	0.00–2.19	0.379
**ACS cVEMP**					
BPPV	0.55	0.23–1.32	0.090	0.02–1.87	0.214
Ménière’s disease	0.65	0.27–1.58	0.272	0.05–2.83	0.487
Head injury/whiplash	0.86	0.36–2.02	0.730	0.05–8.91	1.000
Vestibular neuritis	0.57	0.19–1.72	0.179	0.00–4.56	0.467
Spontaneous vertigo	1.37	0.86–2.17	0.267	0.42–17.56	0.455
Positional vertigo	0.66	0.22–2.00	0.409	0.03–4.51	0.716
**BCV oVEMP**					
BPPV	0	-	0.003 *	0.00–0.34	0.004 *
Ménière’s disease	1.20	0.43–3.35	0.716	0.17–9.31	1.000
Head injury/whiplash	1.58	0.57–4.39	0.430	0.17–39.89	0.783
Vestibular neuritis	1.67	0.61–4.56	0.343	0.19–188.69	0.691
Spontaneous vertigo	0.17	0.02–1.48	0.037 *	0.00–1.14	0.073
Positional vertigo	2.20	0.85–5.71	0.166	0.36–45.47	0.365
**vHIT (LSCC)**					
BPPV	0	-	0.156	0.00–2.60	0.416
Ménière’s disease	0.97	0.13–7.56	0.980	0.01–13.90	1.000
Vestibular neuritis	1.63	0.26–10.14	0.595	0.09–38.03	1.000
Spontaneous vertigo	1.13	0.24–5.47	0.871	0.09–10.32	1.000
Positional vertigo	0	-	0.281	0.00–4.30	0.740
**vHIT (ASCC)**					
BPPV	3.31	1.12–9.78	0.041 *	0.68–78.76	0.126
Ménière’s disease	0.85	0.26–2.80	0.793	0.04–9.32	1.000
Vestibular neuritis	0.93	0.37–2.33	0.904	0.04–20.11	1.000
Spontaneous vertigo	0.22	0.03–1.46	0.047 *	0.00–1.38	0.106
Positional vertigo	1.28	0.45–3.61	0.691	0.08–26.86	1.000
**vHIT (PSCC)**					
BPPV	1.63	0.78–3.42	0.274	0.31–35.56	0.512
Ménière’s disease	1.10	0.34–3.54	0.880	0.08–12.37	1.000
Vestibular neuritis	1.11	0.18–6.86	0.904	0.05–25.21	1.000
Spontaneous vertigo	0.29	0.04–2.25	0.171	0.00–2.50	0.359
Positional vertigo	1.52	0.39–5.99	0.552	0.12–27.91	0.920

Abbreviations: PPPD = persistent postural-perceptual dizziness, BPPV = benign paroxysmal positional vertigo, ACS cVEMP = cervical vestibular evoked, myogenic potential testing to air-conducted sound, BCV oVEMP = ocular vestibular evoked myogenic potential testing to bone-conducted vibration, vHIT = video head impulse test, LSCC = lateral semicircular canal, ASCC = anterior semicircular canal, PSCC = posterior semicircular canal, CMH = Cochran–Mantel–Haenszel test. * <0.05.

## Data Availability

The raw data supporting the conclusions of this article will be made available by the authors, without undue reservation.
